# Estradiol modulates neural response to conspecific and heterospecific song in female house sparrows: An *in vivo* positron emission tomography study

**DOI:** 10.1371/journal.pone.0182875

**Published:** 2017-08-23

**Authors:** Christine R. Lattin, Frank A. Stabile, Richard E. Carson

**Affiliations:** 1 Department of Radiology and Biomedical Imaging, Yale Positron Emission Tomography Center, Yale University, New Haven, Connecticut, United States of America; 2 Department of Ecology and Evolutionary Biology, Yale University, New Haven, Connecticut, United States of America; Texas Christian University, UNITED STATES

## Abstract

Although there is growing evidence that estradiol modulates female perception of male sexual signals, relatively little research has focused on female auditory processing. We used *in vivo* ^18^F-fluorodeoxyglucose (^18^F-FDG) positron emission tomography (PET) imaging to examine the neuronal effects of estradiol and conspecific song in female house sparrows (*Passer domesticus*). We assessed brain glucose metabolism, a measure of neuronal activity, in females with empty implants, estradiol implants, and empty implants ~1 month after estradiol implant removal. Females were exposed to conspecific or heterospecific songs immediately prior to imaging. The activity of brain regions involved in auditory perception did not differ between females with empty implants exposed to conspecific vs. heterospecific song, but neuronal activity was significantly reduced in females with estradiol implants exposed to heterospecific song. Furthermore, our within-individual design revealed that changes in brain activity due to high estradiol were actually greater several weeks after peak hormone exposure. Overall, this study demonstrates that PET imaging is a powerful tool for assessing large-scale changes in brain activity in living songbirds, and suggests that after breeding is done, specific environmental and physiological cues are necessary for estradiol-stimulated females to lose the selectivity they display in neural response to conspecific song.

## Introduction

For many animals, seasonal variation in steroid hormone production helps coordinate reproductive behavior between signal producers and receivers, partly by altering sensory processing. For example, estradiol increases the range of frequency sensitivity in the hearing organs of female midshipman fish (*Porichthys notatus*), causing enhanced perception of the dominant frequencies of male vocalizations [1]. Although female birds also use vocalizations to find and choose mates, most research on the role of estradiol in avian acoustic communication has focused on its effects on vocal signal production and perception in males (e.g. [2, 3]). The few studies conducted in female birds suggest that estradiol plays an important role in modulating female perception of male acoustic signals. For example, female white-throated sparrows (*Zonotrichia albicollis*) treated with estradiol show increased immediate early gene expression in auditory processing centers of the brain after hearing conspecific song, but not after hearing frequency-matched tones [4–6].

Many techniques for assessing brain responses to different stimuli either require euthanizing animals (e.g., immediate early gene expression methods) or restrict analysis to a small area of the brain (e.g., electrophysiological recordings). Positron emission tomography (PET) overcomes these limitations through *in vivo* functional imaging of the whole brain using molecules labeled with radionuclides with short half-lives, like ^18^F (t_1/2_ = 110 min). Using the glucose analogue ^18^F-fluorodeoxyglucose (^18^F-FDG), it is possible to examine glucose metabolism as a measure of neuronal activity [7]. An advantage of PET over magnetic resonance imaging (MRI), another *in vivo* imaging technology, is that ^18^F-FDG can be injected into awake, freely-behaving animals, whereas most animals must be anesthetized to undergo MRI imaging. After the uptake phase, when ^18^F-FDG is predominantly fixed in the most metabolically active parts of the brain, animals can be anesthetized and imaged. The PET data are then used to construct three-dimensional images depicting the distribution of radioactivity––and thus, integrated neuronal activity––throughout the brain.

Advances in detector technology make PET an increasingly attractive method for small animal brain imaging [8]. However, resolution issues persist, and the highest resolution possible with current microPET scanners and iterative reconstruction algorithms is on the order of ~1 mm [9]. Recent studies using ^18^F-FDG PET in wild crows revealed brain areas associated with threatening and non-threatening stimuli [10, 11], but it is unclear if PET resolution is sufficient to assess brain responses in smaller birds. Furthermore, to our knowledge, no study in any wild animal species has exploited one of the major potential advantages of *in vivo* PET imaging––the ability to perform multiple scans with the same animals. This repeatability provides an opportunity to uncover individual variation in the subtle connections between different stimuli and brain responses and to evaluate how these responses may change over time.

In this study, we used ^18^F-FDG PET in female house sparrows (*Passer domesticus*; n = 10) to assess neuronal activity in response to conspecific and heterospecific song with empty implants, estradiol implants, and empty implants ~1 month after estradiol implant removal. Each female was imaged multiple times with different implant types and song exposures. We predicted that estradiol-treated females exposed to conspecific song would display increased activity in six brain areas containing regions known to be involved in song recognition and auditory processing.

## Materials and methods

### Study subjects and hormonal manipulation

Female house sparrows (n = 10) were caught on 23 Nov 2015 using mist nets and Potter traps at bird feeders in New Haven and Branford, CT, USA. In temperate regions, house sparrows are no longer photorefractory by late autumn [12], so females should be responsive to hormone treatment at this time. In the lab, females were singly housed with *ad libitum* access to mixed seeds, grit and water. Day length in the lab corresponded to natural day length (10L:14D). This study was carried out in accordance with the Association for the Study of Animal Behaviour/Animal Behavior Society Guidelines for the Use of Animals in Research. Animals were collected under Connecticut state permit 1417011, and all procedures approved by the Yale University Animal Care and Use Committee under permit 2014–11648.

**Fig 1:**
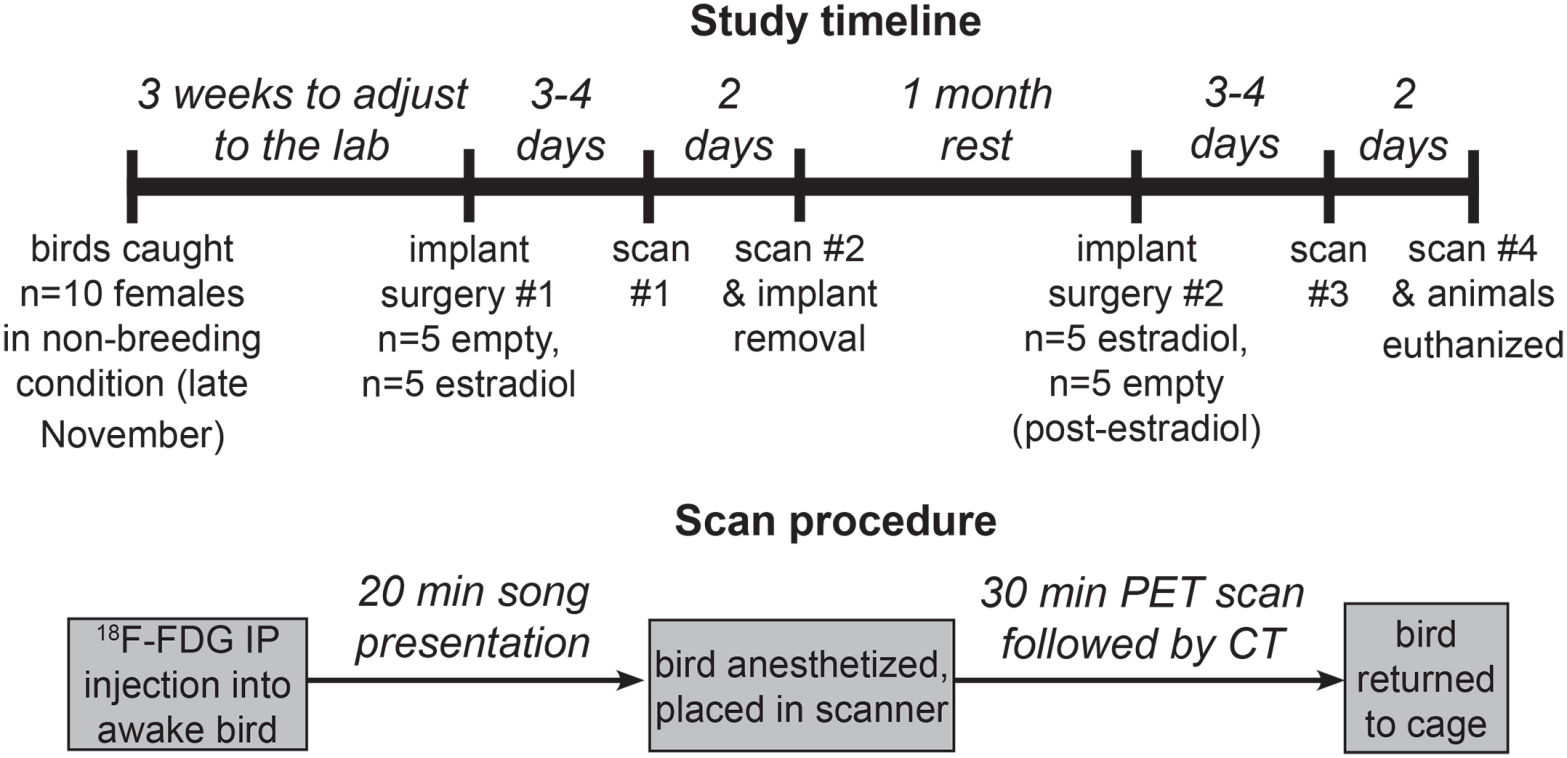
Study design. Top: In a repeated-measures design, female house sparrows (n = 10) received either an empty or estradiol implant and underwent two imaging sessions. Implants were then removed, and after a month, birds received the other implant type and underwent two more imaging sessions. Bottom: Before each imaging session, a female sparrow was given an intraperitoneal (IP) injection of ^18^F-fluorodeoxyglucose (^18^F-FDG) while awake and then exposed to 20 min of song from either male house sparrows or white-throated sparrows.

Three weeks after capture, we inserted subcutaneous implants in all females (Fig 1). Estradiol implants (n = 5) consisted of silastic medical-grade tubing packed with crystalline 17-beta-estradiol (Sigma-Aldrich, St. Louis, MO, USA) and sealed with a silicone adhesive at both ends. Control implants (n = 5) were empty. Implant size (15 mm long, 2 mm outer diameter) was similar to implants used in previous experiments, which produced circulating estradiol concentrations sufficient to elicit breeding behavior in female house sparrows [13]. Animals were anesthetized using inhaled isoflurane (4–5%) and kept at a depth of anesthesia (2.5–4%) at which they did not respond to toe pinch. A small incision was made in cleaned and disinfected skin between the shoulder blades, an implant inserted into the hole, and the incision closed with tissue adhesive (Vetbond, 3M, Maplewood, MN, USA). Females were implanted 3–4 days before the start of stimulus testing, and implants remained in place for 1 week. The surgical sites were monitored throughout the experiments to ensure proper healing and check implant placement. After the first two stimulus tests and imaging sessions, implants were removed. One month later, we performed a second implant surgery where birds that had previously received empty implants received estradiol implants, and vice-versa. After the last two stimulus trials and imaging sessions, birds were euthanized.

### Testing procedure and PET scanning

During stimulus trials, females were exposed to male house sparrow song or male white-throated sparrow song, a control stimulus that most wild house sparrows in this region would have heard. Songs were from the Macaulay Library (Cornell Lab of Ornithology, Ithaca, NY, USA) and the Borror Laboratory of Bioacoustics (The Ohio State University, Columbus, OH, USA). All songs were recorded during the pre-breeding/breeding period (March-July), and singers identified as male by the recordist or the singer's use of male-specific song types [12]. Songs from several males were combined in a randomized order for each playback session to create 40 unique 20 min playlists. During a trial, a female heard song from ~20 different male house sparrows or white-throated sparrows, and each female heard new songs during every trial. All females heard both song types with both implant types, and the order of treatments was randomized for each female and balanced across implant types. The loudness of stimuli presented (in dB) was standardized across all treatments.

To prepare for imaging, all birds were fasted overnight to avoid the risk of animals aspirating food under anesthesia and to reduce circulating plasma glucose that competes with ^18^F-FDG in the brain [14]. Before each stimulus trial, we weighed each bird and took ~120 ul of blood from the brachial vein using a 26-gauge needle for glucose and estradiol assays. We administered intraperitoneal (IP) injections of ~0.4 mCi of ^18^F-FDG (dose range: 0.29–0.57 mCi; volume range: 50–200 ul) to awake females. Females were then exposed to a song stimulus for 20 min and their behavior video-recorded. Previous studies found that estradiol-treated female songbirds may display copulation solicitation behavior in response to conspecific song [15], but in this study we found no significant effects of hormone treatment or song type on any behaviors (S1 Table) so behavior will not be reported further. In pilot scans, we found that after IP injection peak concentrations of ^18^F-FDG occur in the brain at 20 min (S1 Fig), similar to the ~25 min it took for ^18^F-FDG activity to peak in crow brain [10].

After the trial, each bird was anesthetized using isoflurane and imaged in an Inveon microPET-CT scanner (Siemens Medical Solutions USA, Inc., Malvern, PA, USA), as described previously [16]. We imaged the sparrow's whole body using a 30 min PET scan followed by a ~7 min CT scan (for attenuation correction and defining regions of interest). For each female, the two sessions were 72 h (first two scans) or 48 h (second two scans) apart. PET data were reconstructed for the entire 30 min time frame using a vendor-supplied 3D OSEM/MAP algorithm with CT attenuation and scatter correction applied to the data (S2 Fig). We used 2 OSEM iterations, 16 subsets, 18 MAP iterations, and a beta of 0.0023, identified as providing full width at half maximum resolution of 0.8 mm.

### Estradiol implant validation and plasma glucose measures

After centrifugation at 10,000 g, plasma was removed and stored at -80°C. For estradiol assays, we first used solid phase extraction (Supelco Visiprep system, Sigma-Aldrich, St. Louis, MO, USA) to separate polar and non-polar plasma fractions, using 30 ul of plasma diluted in 220 ul of distilled water [17]. We used an enzyme-linked immunoassay (Cayman Chemical Company, Ann Arbor, MI, USA) to measure estradiol. All samples were run in duplicate (mean coefficient of variation between duplicates: 4%). Although we did not correct for extraction efficiency, we do not expect any bias in extraction across different implant groups because our samples were completely mixed within the assay. Furthermore, extraction efficiency for this assay is typically high and consistent among samples (L. Remage-Healey, personal communication). Assay sensitivity was ~15 pg/ml. We also dissected and weighed ovary tissue after euthanasia. These ovary masses only reflected an animal's physiological response to the final treatment females received (estradiol vs. post-estradiol birds). The person doing the dissections (CRL) was blinded to treatment.

Plasma glucose was assessed using a hand-held glucometer (OneTouch UltraMini, LifeScan Inc., Milpitas CA, USA) with ~3 ul plasma applied to test strips. Although hand-held glucometers designed for human use typically underestimate avian blood glucose relative to published reference values, these devices demonstrate high repeatability and measures from the same instrument can be meaningfully compared [18]. Glucose was measured in duplicate and the two readings averaged (mean coefficient of variation: 4%).

### Image analysis and validations

**Fig 2:**
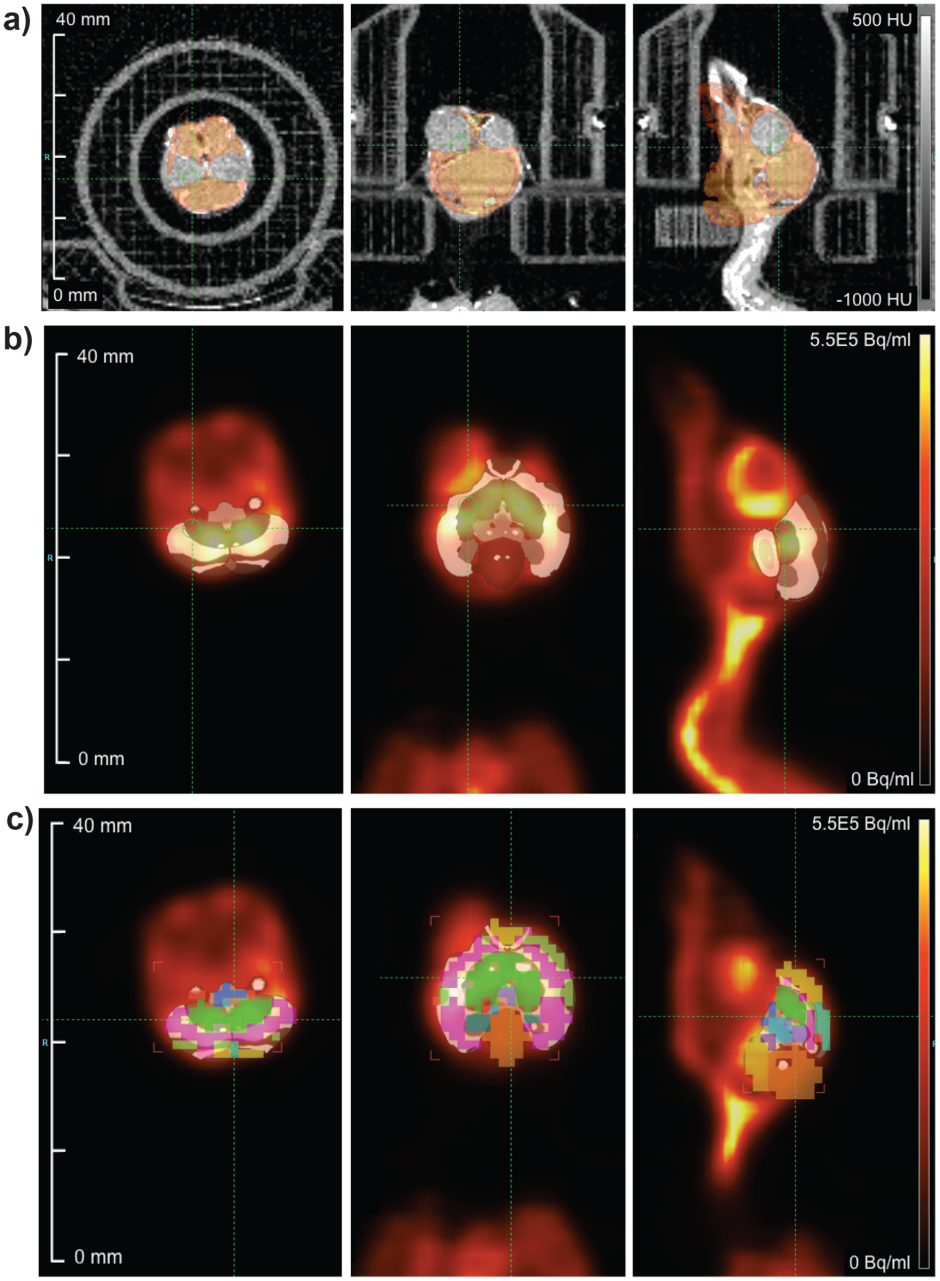
Positron emission tomography (PET) images of female house sparrow brain uptake of ^18^F-fluorodeoxyglucose. Images were processed as follows: a) Computed tomography (CT) images from a published canary atlas were registered with house sparrow CT images using software registration tools. b) Canary atlas regions of interest were transformed to the co-registered sparrow CT and positron emission tomography (PET) images. c) Sparrow regions of interest were created using regions of interest from the canary atlas.

Images were analyzed using Inveon Acquisition Workplace software, version 2.0 (Siemens Healthcare GmbH, Erlangen, Germany). Because there is no published stereotaxic atlas of the house sparrow brain, we used a digital atlas of canary brain [19] to draw regions of interest (ROIs). This atlas divides the brain into 15 regions of interest; however, we restricted our analyses to regions containing areas known to be involved in song recognition and auditory processing: arcopallium (which contains the robust nucleus of the arcopallium), mesopallium (caudal lateral and medial mesopallium), midbrain (lateral mesencephalic nucleus), nidopallium (caudal medial nidopallium, field L, HVC, interfacial nucleus of the nidopallium, and lateral magnocellular nucleus of the anterior nidopallium), striatum (Area X), and thalamus (nucleus ovoidalis) [20–23]. CT images of canary and house sparrow skull were aligned using automated rigid registration tools (Fig 2a). Canary atlas ROIs were then brought into alignment with co-registered sparrow PET-CT images (Fig 2b), and applied to the sparrow PET image (Fig 2c). Note that canary and house sparrow brain volumes are similar (male canary: ~650 mm^3^; female house sparrow: ~700 mm^3^), and use of this canary atlas was validated using a radiotracer that binds specifically only to D_2_ dopamine receptors in the striatum (S3 Fig).

Most ^18^F-FDG PET data are reported as standardized uptake values (SUV), a semiquantitative unitless measure of normalized radioactivity concentration in PET images [24]:

$$SUV=\frac{activity\;concentration\;in\;region\;of\;interest}{\frac{injected\;activity}{body\;mass}}$$

If activity is uniformly distributed in the bird, SUV = 1. Although plasma glucose concentrations can affect tissue uptake of ^18^F-FDG [14], there is debate about whether plasma glucose normalization of SUV is valuable [24, 25]. Because hormone treatment significantly affected plasma glucose concentrations (see Results), we report results both with and without glucose normalization of SUV data. Glucose normalization was performed by multiplying SUV by plasma glucose [26].

IP injections can be highly variable, with different amounts of injected material reaching the bloodstream depending on the exact location of the injection and the uptake of nearby tissues [27]. Lung SUV was not affected by hormone treatment, song type, scan number, or hormone treatment x song type interactions (S2 Table). Therefore, we also explored normalizing brain SUV by lung SUV:

$$normalized\;brain\;SUV=\frac{brain\;SUV}{lung\;SUV}$$

Lung SUV values were scaled based on the lower density of these tissues, as measured in the CT images, as follows [28]:

$$density\text{-}scaled\;SUV=\frac{tissue\;attenuation\;of\;lung\;in\;Hounsfield\;units+1000}{tissue\;attenuation\;of\;muscle\;in\;Hounsfield\;units+1000}$$

The activity in the lung was divided by this scaling factor, and density-scaled activity used to calculate SUV.

### Statistical analysis

All analyses were performed in JMP Pro 11. Birds that received estradiol implants before empty implants had slightly elevated plasma estradiol concentrations 1 month later (see Results), so we analyzed these birds as a third group called post-estradiol. We also explored potential differences in the neural responses of females receiving estradiol implants first vs. second, but found no response differences between these groups (estradiol 1 vs. estradiol 2: t = 1.3, df = 11.5, p = 0.20), so all birds with estradiol implants were combined for the final analysis. Therefore, final samples sizes for each group were: empty: n = 5, estradiol: n = 10, post-estradiol: n = 5. For all repeated-measures analyses (which included SUV, glucose-normalized SUV, and lung-normalized SUV as dependent variables), we used linear mixed models based on a restricted maximum likelihood approach, with hormone treatment (empty implant, estradiol implant, or post-estradiol), brain region (arcopallium, mesopallium, midbrain, nidopallium, striatum, and thalamus), song type (conspecific or heterospecific), scan number (1, 2, 3, or 4) and hormone treatment x song type interactions as fixed effects and individual bird as a random effect to account for the repeated-measures nature of the data [29]. We did not have sufficient statistical power to include brain region x song type, brain region x hormone treatment, or brain region x song type x hormone treatment effects in our full model. However, we did perform an exploratory analysis comparing SUV of nidopallium, which contains several regions expected to be responsive to auditory stimuli, and cerebellum, which does not. The plasma glucose analysis was a simplified version of our full model, with hormone treatment and scan number as fixed effects and individual bird as a random effect. Ovary mass from females at the end of the study was compared using Welch's ANOVA because of unequal variances between the two groups. We checked for homoscedasticity by inspecting plots of studentized residuals against predicted values of dependent variables. Where appropriate, we used Student's t-tests as post-hoc tests with planned comparisons to minimize the risk of committing Type I error. All measures are presented as means ± SEM.

## Results

Birds with estradiol implants had higher circulating estradiol (2.5 ± 0.7 ng/ml) than birds in the empty (0.16 ± 0.05 ng/ml) and post-estradiol (1.1 ± 0.8 ng/ml) groups. A mixed model analysis revealed a significant hormone treatment effect on plasma estradiol (F_2,10_ = 6.6, p = 0.016), and post-hoc tests revealed that birds with estradiol implants had significantly higher plasma hormone titers than birds in the other two groups (empty vs. estradiol: t = -2.8, df = 9.7, p = 0.019; post-estradiol vs. estradiol: t = -2.4, df = 9.7, p = 0.039; post-estradiol vs. empty: t = 0.3, df = 11.8, p = 0.76). At the end of the study, ovary mass from females with estradiol implants was significantly greater than post-estradiol females with empty implants (estradiol: 437.6 ± 102.5 mg; post-estradiol: 13.4 ± 12.3 mg; F_1,4_ = 4.02, p = 0.015). There was also a significant hormone treatment effect on plasma glucose concentrations (empty implant: 490.9 ± 9.1 mg/dl; estradiol implant: 468.8 ± 11.3 mg/dl; post-estradiol: 508.3 ± 10.5 mg/dl; F_2,19_ = 10.9 p = 0.0007). Post-hoc tests revealed that plasma glucose of post-estradiol birds was higher than birds in the empty and estradiol implant groups (estradiol vs. empty: t = -1.3, df = 32.9, p = 0.19; post-estradiol vs. empty: t = 2.8, df = 10.1, p = 0.018; post-estradiol vs. estradiol: t = 4.7, df = 32.9, p < 0.0001).

**Fig 3:**
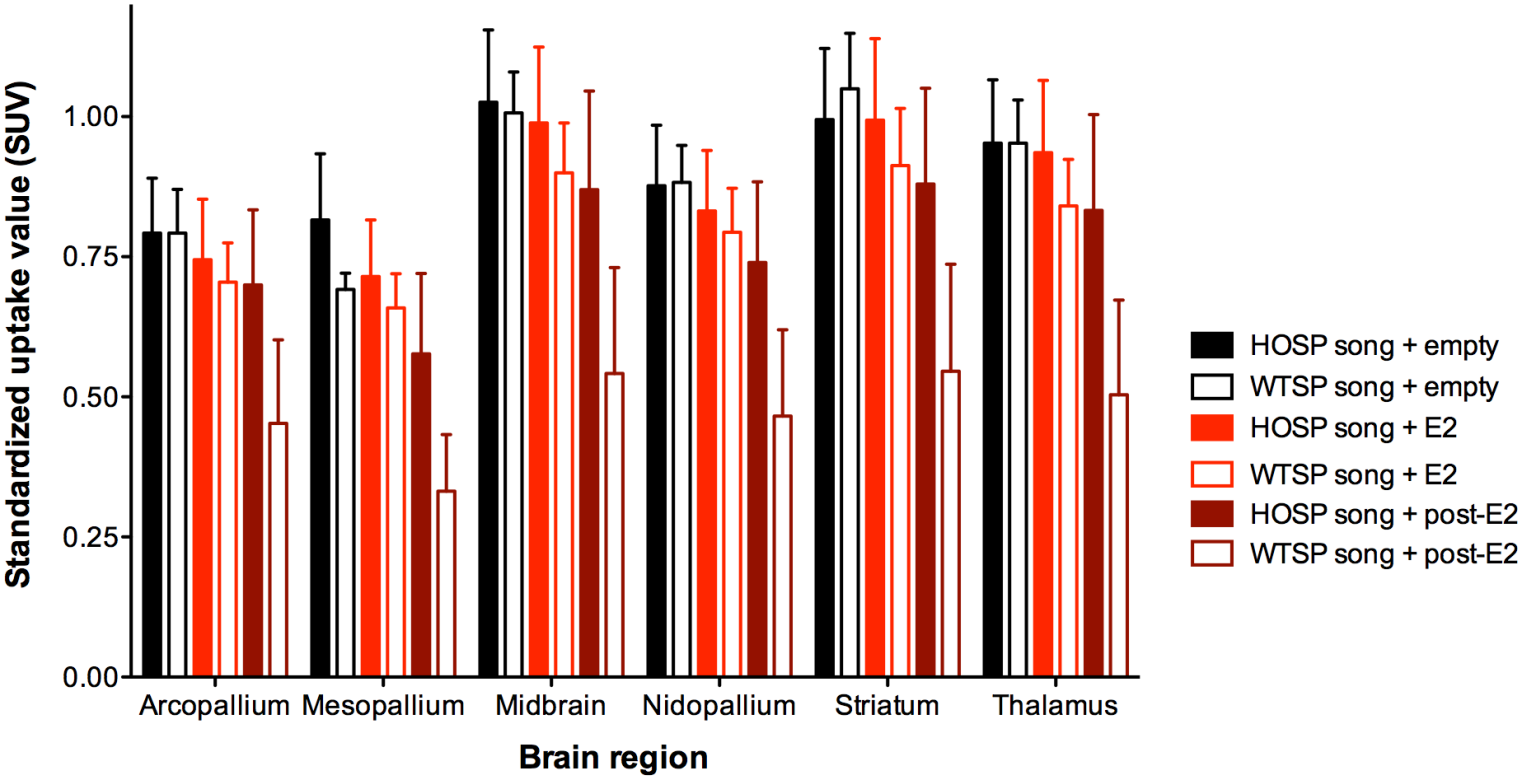
Standardized uptake values (SUV) of ^18^F-fluorodeoxyglucose in six brain regions in house sparrow females with empty implants (n = 5), estradiol implants (E2, n = 10), or empty implants ~1 month after having an estradiol implant (post-E2; n = 5) in response to house sparrow (HOSP) or white-throated sparrow (WTSP) song. These six regions were included in our analysis because they contain areas previously associated with auditory perception and song recognition. See Table 1 for statistical results from linear mixed models.

SUV of ^18^F-FDG in arcopallium, mesopallium, midbrain, nidopallium, striatum, and thalamus was significantly affected by song type, hormone treatment, and brain region, and there was a significant song type x hormone treatment interaction (Table 1, Fig 3). Normalizing brain SUV data using plasma glucose or lung SUV did not substantially change the results of our analysis, except that hormone treatment was no longer significant when brain SUV was scaled by plasma glucose or by lung SUV (Table 1), suggesting that hormone treatment may have affected ^18^F-FDG bioavailability. Furthermore, despite a significant treatment effect in the main model for non-normalized SUV (Table 1), post-hoc testing did not reveal any significant differences between birds with empty implants, estradiol implants, or in birds with empty implants ~1 month after estradiol treatment (estradiol vs. empty: t = 0.7, df = 189.7, p = 0.47; post-estradiol vs. empty: t = -0.5, df = 178.1, p = 0.64; post-estradiol vs. estradiol: t = -1.6, df = 191.4, p = 0.11.) Song type effects and song type x hormone treatment interactions remained significant by all analysis methods.

**Table 1:** Effects of hormone treatment, brain region, song type, scan number and hormone treatment x song type interactions on ^18^F-fluorodeoxyglucose standardized uptake values (SUV) in female house sparrow brain (*Passer domesticus*). Results are from linear mixed models with individual bird as a random effect. To account for differences in plasma glucose concentrations and injection efficiency, we also performed analyses on glucose-normalized and lung-normalized SUV. See text for more details on normalization. Significant model effects are in bold.

Model effect	SUV	Glucose-normalized SUV	Lung-normalized SUV
Hormone treatment	F_2,205_ = 5.7, **p = 0.0038**	F_2,203_ = 0.07, p = 0.93	F_2,160_ = 2.32, p = 0.10
Brain region	F_5,213_ = 15.3, **p < 0.0001**	F_5,213_ = 13.9, **p < 0.0001**	F_5,213_ = 21.4, **p < 0.0001**
Song type	F_1,215_ = 25.5, **p < 0.0001**	F_1,215_ = 27.0, **p < 0.0001**	F_1,217_ = 16.9, **p < 0.0001**
Scan number	F_1,193_ = 0.26, p = 0.61	F_1,189_ = 1.97, p = 0.16	F_1,130_ = 1.26, p = 0.26
Hormone treatment x song type	F_2,219_ = 8.0, **p = 0.0004**	F_2,219_ = 8.0, **p = 0.0005**	F_2,217_ = 11.0, **p < 0.0001**

**Fig 4:**
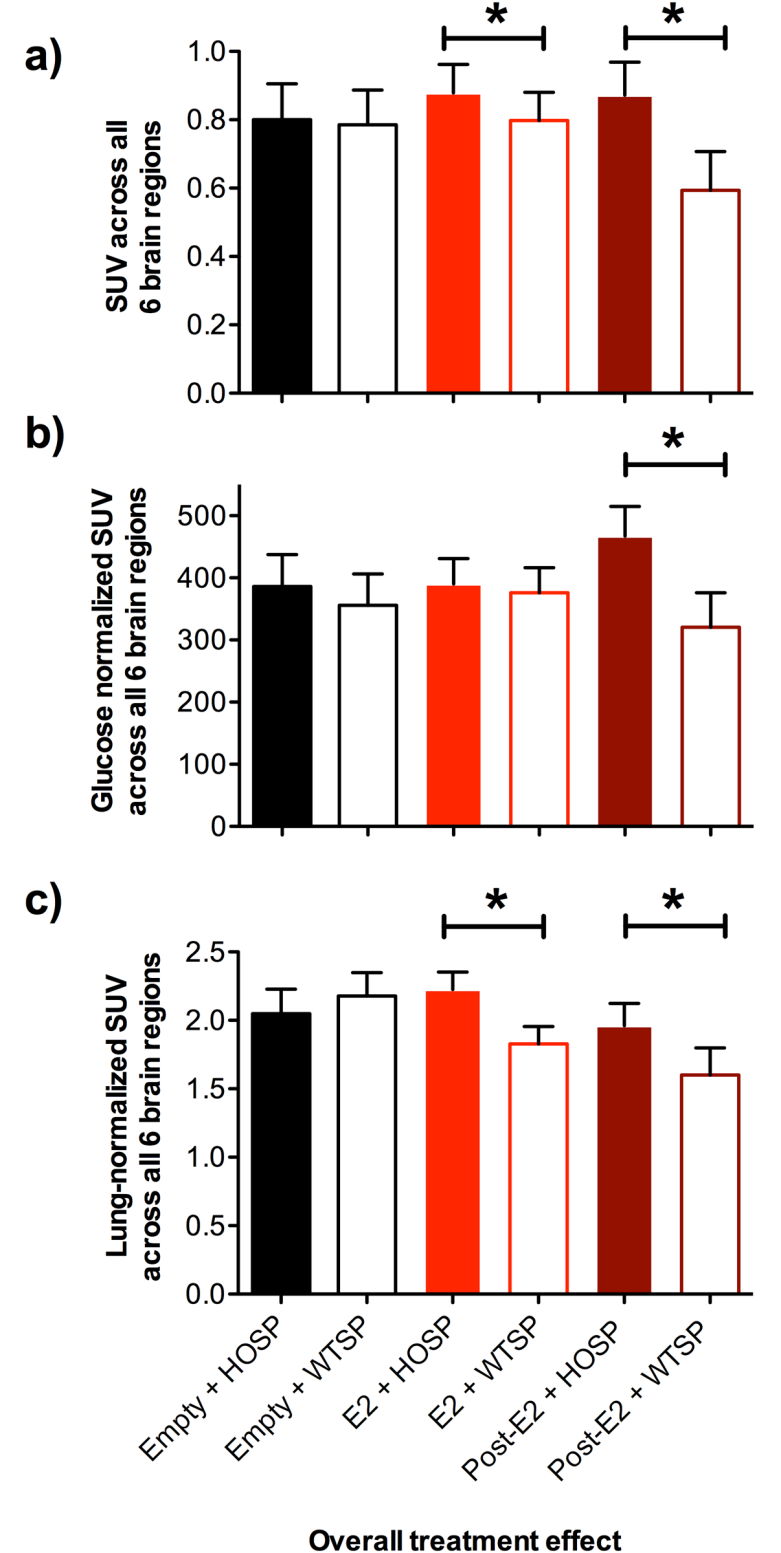
Standardized uptake values (SUV) of ^18^F-fluorodeoxyglucose in six brain regions involved in auditory processing in house sparrow females with empty implants (black bars; n = 5), estradiol implants (E2, light red; n = 10) and empty implants ~1 month after having an estradiol implant (post-E2, dark red bars; n = 5). Females were exposed to the song of breeding male house sparrows (HOSP, filled bars) or white-throated sparrows (WTSP, empty bars). a) non-normalized SUV, b) SUV normalized using plasma glucose concentrations, c) SUV normalized by lung SUV. Stars indicate significant differences in SUV between song types within a treatment, as indicated by post-hoc tests.

For song type x hormone treatment interactions, we compared SUV of house sparrow song vs. white-throated sparrow song within each hormone treatment (Fig 4). For the post-estradiol group, brain ^18^F-FDG uptake was lower after exposure to white-throated sparrow song regardless of whether or not SUV was normalized by plasma glucose or lung SUV (non-normalized SUV: t = -5.2, df = 222, p < 0.0001; glucose-normalized SUV: t = -5.5, df = 222, p < 0.0001; lung-normalized SUV: t = -3.3, df = 221, p = 0.001). For the estradiol group, only non-normalized brain SUV and lung-normalized brain SUV showed decreased ^18^F-FDG uptake in response to heterospecific song (non-normalized SUV: t = -2.2, df = 221, p = 0.029; glucose-normalized SUV: t = -0.8, df = 221, p = 0.44; lung-normalized SUV: t = -5.2, df = 222, p < 0.0001). For the empty group, there were no differences in brain response to the two different song types regardless of whether or not SUV was normalized by glucose or lung SUV (non-normalized SUV: t = -0.2, df = 214, p = 0.81; glucose-normalized SUV: t = -1.3, df = 214, p = 0.18; lung-normalized SUV: t = 1.4, df = 214, p = 0.18).

An exploratory analysis of cerebellum vs. nidopallium did not reveal significant brain region x song type, brain region x hormone treatment, or brain region x song type x hormone treatment interactions (S3 Table). The pattern of ^18^F-FDG uptake in the two brain regions was very similar, as it was across all brain regions, including regions we did not include in our analysis (Fig 3, S4 Fig).

## Discussion

In female house sparrows exposed to conspecific or heterospecific songs, we found that estradiol treatment significantly affected the uptake of ^18^F-FDG in six brain regions containing areas involved in song recognition and auditory processing: arcopallium, mesopallium, midbrain, nidopallium, striatum, and thalamus. More specifically, neuronal responses to the two song types did not differ in females with empty implants, but ^18^F-FDG uptake was significantly reduced in females exposed to heterospecific song in the post-estradiol group (for all quantification approaches) and the estradiol group (for non-normalized and lung-normalized SUV models). To our knowledge, this is the first *in vivo* study demonstrating widespread estradiol-induced changes in brain responses to environmentally relevant auditory stimuli, adding to a growing body of literature demonstrating estradiol effects on female perception of male sexual signals [1, 5, 30, 31], even in a species with a relatively simple song type.

The largest difference in ^18^F-FDG uptake (~30%) between the two song types was seen in post-estradiol birds, which received hormone implants ~1 month prior to testing, and had regressed ovaries and relatively low estradiol titers (1.1 ± 0.8 ng/ml), rather than in estradiol-treated birds, which received hormone implants 3–7 days prior to testing, and had large ovaries and high plasma estradiol (2.5 ± 0.7 ng/ml). It should be noted that plasma estradiol in our post-estradiol group was still much higher than in our empty implant group (0.16 ± 0.05 ng/ml, similar to that reported for non-breeding female house sparrows in a previous study [32]). We have no reason to think birds were photostimulated, as they were kept on a constant light cycle corresponding to their capture date in November. However, we do have evidence from animals euthanized at the end of the study that estradiol implants caused ovary growth. Females that received estradiol implants first likely also had a corresponding increase in ovary size, which might have lasted for days or even weeks after implants were removed. Future studies should try to distinguish whether the long-term effects of estradiol on neural responses that we saw are due to exogenous or endogenous hormone. In any case, this result suggests that estradiol's effects on female sensory processing may take more than a few days to fully develop, and that these effects can be relatively long lasting.

The physiological mechanism behind the differences in neuronal response remains unclear. Estradiol may affect the ear directly, stimulate steroid-sensitive brain nuclei, or both. In female zebra finches, both hair cells and the supporting cells of the ear display estrogen receptor immunoreactivity [33]. Estrogen receptors also occur in high density in several parts of the forebrain (including the robust nucleus of the arcopallium and HVC), the striatum, and several other areas of house sparrow brain in both males and females [34]. One possibility is that estradiol causes widespread structural changes across the female songbird brain, similar to the effects of testosterone on the brain of male songbirds [35].

Because using ^18^F-FDG SUV to quantify neuronal response is a relatively novel technique in wild species, we explored normalizing ^18^F-FDG data in several ways. This was particularly important because circulating glucose in post-estradiol birds was higher than in birds with estradiol or empty implants, and high plasma glucose can compete with ^18^F-FDG in the brain, causing reduced uptake. Generally speaking, these different normalizations did not substantially alter our results. The one exception was that normalizing SUV by plasma glucose and lung SUV did eliminate the significant effect of hormone treatment on brain uptake of ^18^F-FDG (although importantly not the interaction between hormone treatment and song type). In mammals, estradiol enhances insulin sensitivity [36] and increases glucose transporter expression in brain [37]. Thus, estradiol effects on plasma glucose may have been largely driving the significant hormone treatment effect in the non-normalized SUV model. These results demonstrate that normalizing SUV by glucose concentrations is essential in cases where treatments cause differences in plasma glucose.

Exploratory analyses examining interactions between different brain regions (nidopallium vs. cerebellum), hormone treatments and song types did not identify any region-specific effects, though many songbird studies have demonstrated that various brain areas respond differently to conspecific and heterospecific song and to estradiol [5, 6, 20]. It is possible that the resolution limits of microPET imaging (~1 mm), combined with the use of a brain atlas from a different songbird species, may have prevented detection of fine-grained region-specific effects, especially for a treatment expected to affect several parts of the brain. However, a more intriguing possibility is that the effects of hormone treatment and stimulus type on neuronal glucose utilization may actually be quite widespread throughout the brain. For example, male and female zebra finches showed differences in global auditory evoked responses when exposed to conspecific vs. heterospecific song [38], and, in house sparrows specifically, auditory brainstem responses to tone bursts in the frequency range of conspecific song were greater in spring than in autumn in both males and females [39]. Auditory brainstem responses have also been shown to vary seasonally in several other songbird species [40–42]. This suggests that there may be hormone-driven variation in auditory performance in many songbird species, which could have effects across the whole brain.

## Conclusions

Despite current resolution limitations, this study demonstrates that PET imaging is a valuable technique for assessing large-scale changes in neuronal activity in living songbirds with brains as small as ~700 mm^3^. As scanner technology and reconstruction algorithms continue to improve, increasing resolution will allow for the study of more and more fine-grained effects. Furthermore, our within-individual design revealed that changes in female neuronal response due to high estradiol were actually greater several weeks after the period of peak hormone exposure. Future studies should focus on determining whether estradiol causes volume increases in brain areas involved in auditory processing and social behavior in female birds as testosterone does in males, as well as in elucidating the external environmental cues and internal physiological cues necessary for heterospecific song to regain the normal salience it possesses for females outside of the breeding season.

## Supporting information

S1 Table Effects of hormone treatment, song type, scan number and hormone treatment x song type interaction on 5 different behaviors in female house sparrows (*Passer domesticus*). Results are from linear mixed models with individual bird as a random effect. All behaviors were transformed to increase normality. We measured all behaviors as previously described [16], except for phonotaxis, which was measured using a stopwatch, and defined as the amount of time a bird spent in the half of the cage closest to the speaker. We saw no clear instances of copulation solicitation behavior. All behaviors were scored by the same person (CRL), who was blinded to implant type, song type, and scan number.
(DOCX)

S2 Table Effects of hormone treatment, song type, scan number and hormone treatment x song type interaction on the standardized uptake value (SUV) of ^18^F-fluorodeoxyglucose in lungs of female house sparrows (*Passer domesticus*), with and without glucose normalization (multiplying SUV by plasma glucose). Results are from linear mixed models with individual bird as a random effect.
(DOCX)

S3 Table Results from an exploratory analysis examining the effects of hormone treatment, brain region (cerebellum vs. nidopallium), song type, scan number, and hormone treatment x song type, brain region x song type, brain region x hormone treatment, and brain region x song type x hormone treatment interactions on standardized uptake value (SUV) of ^18^F-fluorodeoxyglucose in female house sparrow brain (*Passer domesticus*). Results are from linear mixed models with individual bird as a random effect. To account for differences in plasma glucose concentrations and injection efficiency, we also ran analyses on glucose-normalized and lung-normalized SUV. See text for more details on normalization. Bold values indicate significant model effects.
(DOCX)

S1 Fig Time-activity curves for brain uptake of ^18^F-fluorodeoxyglucose administered via intraperitoneal (IP) injection in two house sparrows (*Passer domesticus*). Animals were administered IP ^18^F-fluorodeoxyglucose while awake, then immediately anesthetized and imaged for 1 h. Peak brain uptake can be seen ~20 min after injection (gray box).
(TIFF)

S2 Fig Axial (left), coronal (middle), and sagittal (right) views of house sparrow (*Passer domesticus*) brain from positron emission tomography (PET) scans using the dopamine D_2_ receptor antagonist ^11^C-raclopride. PET data are shown with a yellow-black color scale, overlaid onto a gray scale computed tomography (CT) image. Regions of interest (ROIs) were created using the automated procedure described in the Methods with a digital atlas of canary brain. As expected, the ROI for striatum shows high uptake of ^11^C-raclopride indicative of specific binding, with negligible uptake in the cerebellum ROI.
(TIF)

S3 Fig Maximum intensity projection image of a whole-body positron emission tomography (PET) scan in a female house sparrow (*Passer domesticus*). We administered an intraperitoneal (IP) injection of ^18^F-fluorodeoxyglucose to the animal while awake, exposed it to 20 min of conspecific song, then anesthetized it and collected PET data for 30 min. PET data were reconstructed for the entire time frame using a vendor-supplied 3D OSEM/MAP algorithm with computed tomography attenuation and scatter correction applied to the data.
(TIF)

S4 Fig Standardized uptake values (SUV) of ^18^F-fluorodeoxyglucose in five additional brain regions in house sparrow females with empty implants (n = 5), estradiol implants (E2, n = 10), or empty implants ~1 month after having an estradiol implant (post-E2; n = 5) in response to house sparrow (HOSP) or white-throated sparrow (WTSP) song. These regions were not included in our analysis because they do not contain regions previously associated with auditory perception and song recognition. Although we used a canary brain atlas for delineating regions of interest that includes four additional regions (see main text for details), we do not depict these because the regions were either very small (<10 mm^3^; optic chiasm and septum), or are already included in other regions (brain nuclei, lateral ventricle and fiber tracts). SUV patterns in response to different hormone treatments and song types were similar across all brain regions (see also Fig 3).
(TIFF)

S1 File Maximum intensity projection movie of a whole-body positron emission tomography (PET) scan in a female house sparrow (*Passer domesticus*). (MOV)

S2 File Raw data file. (XLSX)
