# Supplementary material for: Estradiol modulates neural response to conspecific and heterospecific song in female house sparrows: An in vivo positron emission tomography study
Source: PLoS One. 2017 Aug 23;12(8):e0182875. doi: 10.1371/journal.pone.0182875 (PMC5568339; doi:10.1371/journal.pone.0182875)
Supplement: S1 Table — Results are from linear mixed models with individual bird as a random effect. All behaviors were transformed to increase normality. We measured all behaviors as previously described [16], except for phonotaxis, which was measured using a stopwatch, and defined as the amount of time a bird spent in the half of the cage closest to the speaker. We saw no clear instances of copulation solicitation behavior. All behaviors were scored by the same person (CRL), who was blinded to implant type, song type, and scan number. (DOCX) [file pone.0182875.s001.docx]

| Model effect | Log hops and flights/min | Logit time near speaker | Log beak wipes/min | Log preens/min | Log feather ruffles/min |
| --- | --- | --- | --- | --- | --- |
| Hormone treatment | F_2,15_ = 0.19  p = 0.83 | F_2,14_ = 0.85  p = 0.45 | F_2,12_ = 0.86  p = 0.45 | F_2,11_ = 0.37  p = 0.70 | F_2,14_ = 0.67  p = 0.53 |
| Song type | F_1,21_ = 0.36  p = 0.56 | F_1,20_ = 0.0097  p = 0.93 | F_1,17_ = 0.0021  p = 0.96 | F_1,18_ = 0.90  p = 0.36 | F_1,19_ = 0.14  p = 0.71 |
| Scan number | F_3,21_ = 0.83  p = 0.49 | F_3,22_ = 0.34  p = 0.80 | F_3,18_ = 2.18  p = 0.13 | F_3,21_ = 1.05  p = 0.39 | F_3,20_ = 1.89  p = 0.16 |
| Hormone treatment x song type | F_2,21_ = 0.52  p = 0.60 | F_2,20_ = 0.71  p = 0.50 | F_2,16_ = 1.53  p = 0.25 | F_2,17_ = 1.15  p = 0.34 | F_2,18_ = 0.85  p = 0.44 |
