# Supplementary material for: Estradiol modulates neural response to conspecific and heterospecific song in female house sparrows: An in vivo positron emission tomography study
Source: PLoS One. 2017 Aug 23;12(8):e0182875. doi: 10.1371/journal.pone.0182875 (PMC5568339; doi:10.1371/journal.pone.0182875)
Supplement: S2 Table — Results are from linear mixed models with individual bird as a random effect. (DOCX) [file pone.0182875.s002.docx]

| **Effect tested** | **Results** | |
| --- | --- | --- |
|  | *without glucose normalization* | *with glucose normalization* |
| Hormone treatment | F_2,15_ = 1.70  p = 0.22 | F_2,15_ = 0.31  p = 0.74 |
| Song type | F_1,22_ = 0.62  p = 0.44 | F_1,22_ = 0.58  p = 0.74 |
| Scan number | F_3,23_ = 0.65  p = 0.59 | F_3,23_ = 0.43  p = 0.73 |
| Hormone treatment x song type | F_2,22_ = 0.70  p = 0.51 | F_2,22_ = 1.18  p = 0.32 |
