# Supplementary material for: Estradiol modulates neural response to conspecific and heterospecific song in female house sparrows: An in vivo positron emission tomography study
Source: PLoS One. 2017 Aug 23;12(8):e0182875. doi: 10.1371/journal.pone.0182875 (PMC5568339; doi:10.1371/journal.pone.0182875)
Supplement: S3 Table — Results are from linear mixed models with individual bird as a random effect. To account for differences in plasma glucose concentrations and injection efficiency, we also ran analyses on glucose-normalized and lung-normalized SUV. See text for more details on normalization. Bold values indicate significant model effects. (DOCX) [file pone.0182875.s003.docx]

| Model effect | SUV | Glucose-normalized SUV | Lung-normalized SUV |
| --- | --- | --- | --- |
| Hormone treatment | F_2,56_ = 2.5  p = 0.093 | F_2,55_ = 0.09  p = 0.91 | F_2,42_ = 1.9  p = 0.17 |
| Brain region | F_1,56_ = 6.8  **p = 0.012** | F_1,56_ = 6.3  **p = 0.015** | F_1,56_ = 10.3  **p = 0.002** |
| Song type | F_1,57_ = 8.1  **p = 0.006** | F_1,57_ = 8.6  **p = 0.005** | F_1,58_ = 5.1  **p = 0.028** |
| Scan number | F_1,54_ = 0.04  p = 0.84 | F_1,52_ = 0.11  p = 0.75 | F_1,39_ = 0.06  p = 0.81 |
| Hormone treatment x song type | F_2,61_ = 3.0  p = 0.055 | F_2,61_ = 3.9  **p = 0.026** | F_2,61_ = 2.1  p = 0.13 |
| Brain region x song type | F_1,56_ = 0.04  p = 0.84 | F_1,56_ = 0.03  p = 0.86 | F_1,57_ = 0.08  p = 0.78 |
| Brain region x hormone treatment | F_2,56_ = 0.18  p = 0.83 | F_2,56_ = 0.15  p = 0.86 | F_2,57_ = 0.27  p = 0.76 |
| Brain region x hormone treatment x song type | F_2,56_ = 0.17  p = 0.85 | F_2,56_ = 0.12  p = 0.89 | F_2,57_ = 0.27  p = 0.76 |
