# Supplementary figures and images for: Estradiol modulates neural response to conspecific and heterospecific song in female house sparrows: An in vivo positron emission tomography study
Source: PLoS One. 2017 Aug 23;12(8):e0182875. doi: 10.1371/journal.pone.0182875 (PMC5568339; doi:10.1371/journal.pone.0182875)

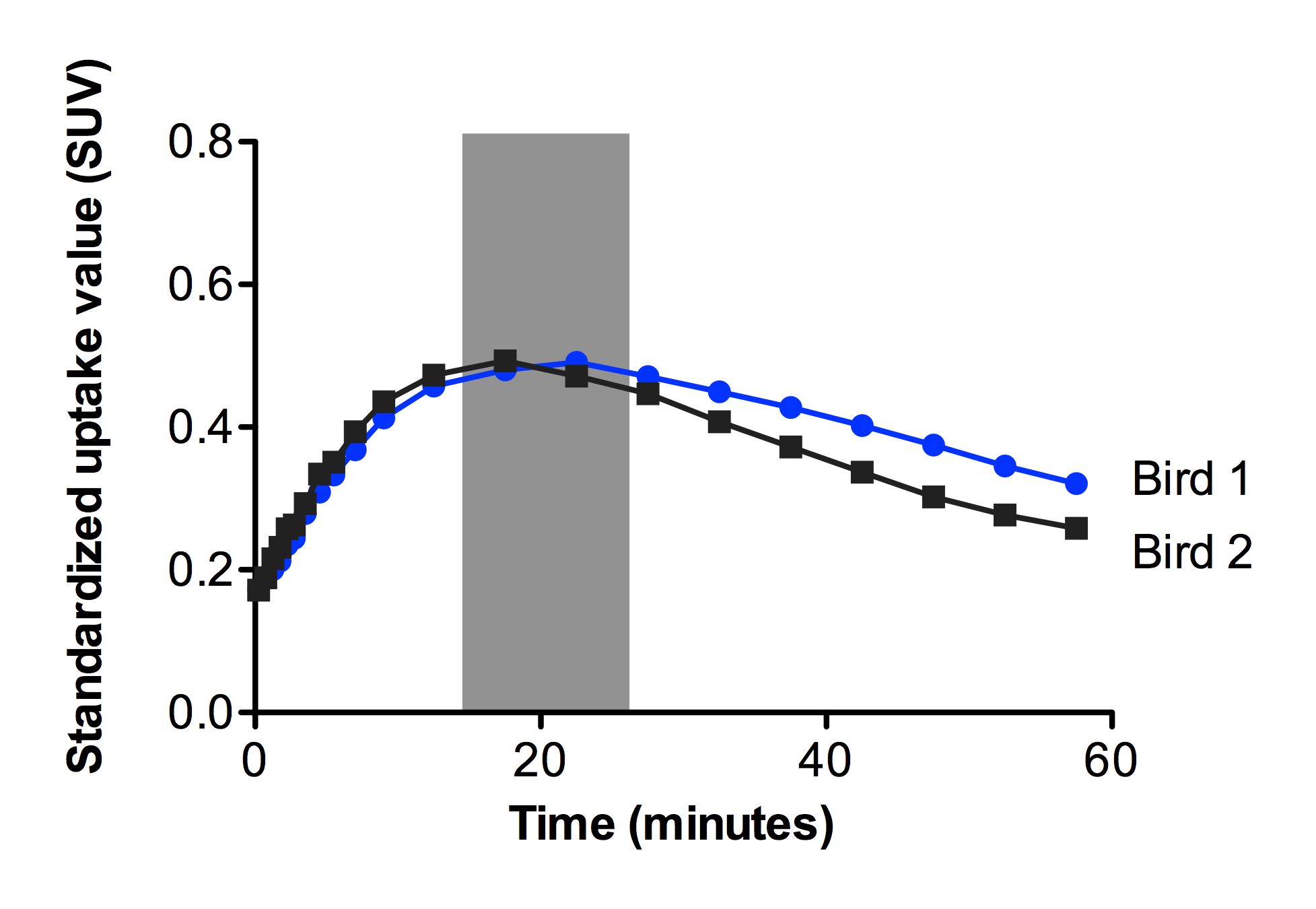

Supplement: S1 Fig — Animals were administered IP 18F-fluorodeoxyglucose while awake, then immediately anesthetized and imaged for 1 h. Peak brain uptake can be seen ~20 min after injection (gray box). (TIFF) [file pone.0182875.s004.tiff]

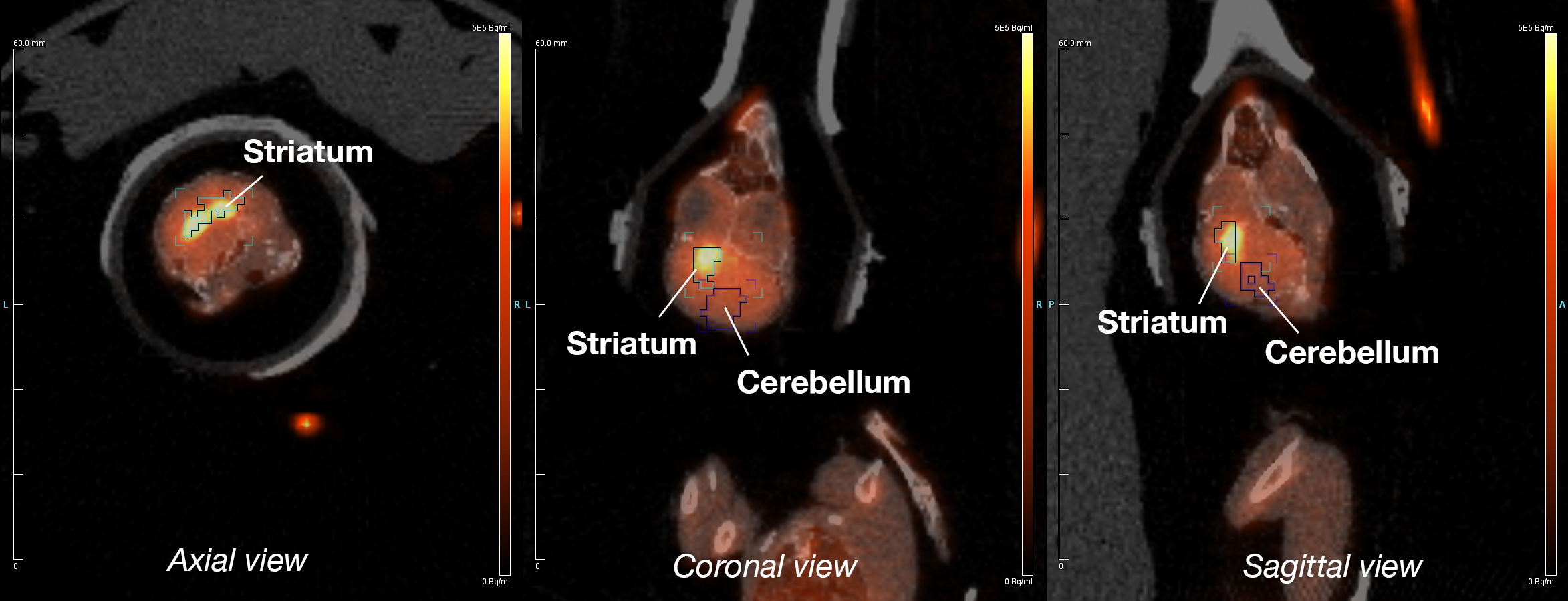

Supplement: S2 Fig — PET data are shown with a yellow-black color scale, overlaid onto a gray scale computed tomography (CT) image. Regions of interest (ROIs) were created using the automated procedure described in the Methods with a digital atlas of canary brain. As expected, the ROI for striatum shows high uptake of 11C-raclopride indicative of specific binding, with negligible uptake in the cerebellum ROI. (TIF) [file pone.0182875.s005.tif]

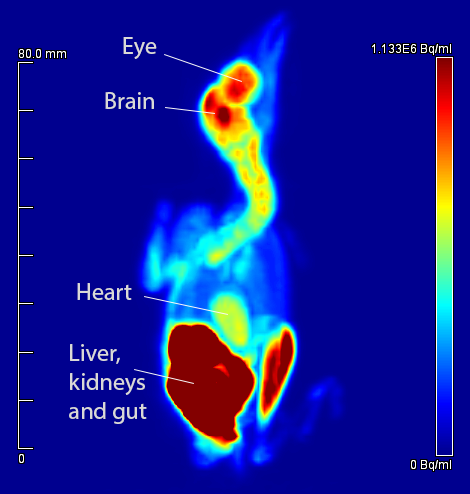

Supplement: S3 Fig — We administered an intraperitoneal (IP) injection of 18F-fluorodeoxyglucose to the animal while awake, exposed it to 20 min of conspecific song, then anesthetized it and collected PET data for 30 min. PET data were reconstructed for the entire time frame using a vendor-supplied 3D OSEM/MAP algorithm with computed tomography attenuation and scatter correction applied to the data. (TIF) [file pone.0182875.s006.tif]

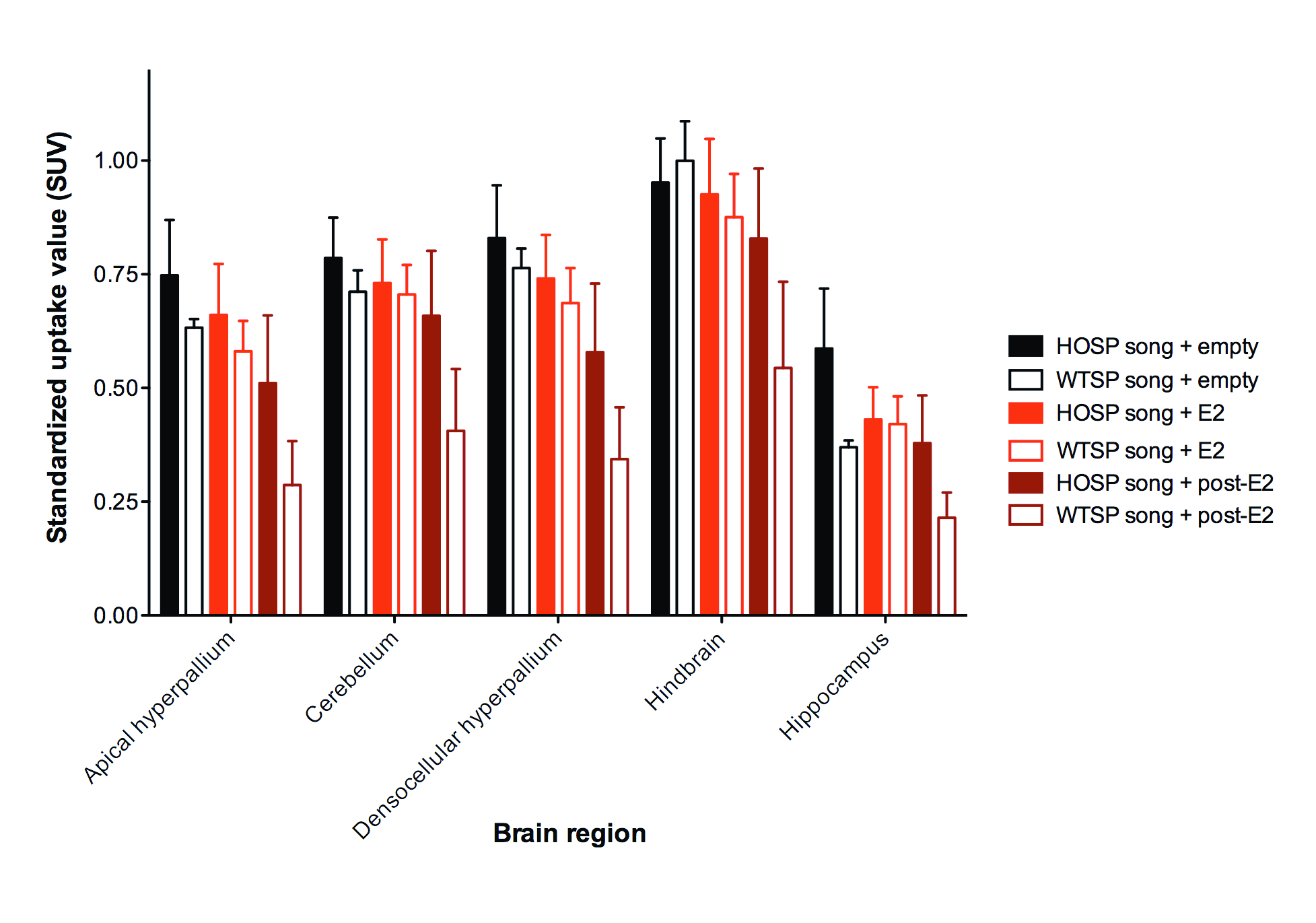

Supplement: S4 Fig — These regions were not included in our analysis because they do not contain regions previously associated with auditory perception and song recognition. Although we used a canary brain atlas for delineating regions of interest that includes four additional regions (see main text for details), we do not depict these because the regions were either very small (<10 mm3; optic chiasm and septum), or are already included in other regions (brain nuclei, lateral ventricle and fiber tracts). SUV patterns in response to different hormone treatments and song types were similar across all brain regions (see also Fig 3). (TIFF) [file pone.0182875.s007.tiff]
